# An American Veterinarian’s Impressions of the Profession in England*Read before the Massachusetts Veterinary Society.

**Published:** 1885-10

**Authors:** Austin Peters


### Art. XXV.—AN AMERICAN VETERINARIAN'S IMPRESSIONS OF THE PROFESSION IN ENGLAND.*

BY AUSTIN PETERS, D.V.S., M.R.C.V.S.

Having been appointed, by our worthy President essayist for this meeting, I thought it might be interesting if I gave a short account of what I saw in England, and the impressions I received regarding the condition of the veterinary profession there.

To criticise, in a friendly way, that which I disapprove of, and to praise that which is worthy of commendation.

I presented myself at the Royal Veterinary College, Camden Town, London, May 3d, 1884, armed with letters of introduction to Prof. Robertson, the Principal, and Prof. Axe. Both these gentlemen received me very cordially and kindly, and the Principal showed me over the institution.

The buildings and grounds of the college must cover nearly an acre; there are accommodations for about one hundred horses, besides room for cattle, sheep and dogs. The Principal's house is among the college buildings, and has a nice little garden.

The museum was rather a disappointment to me. It consisted of only one room, with a gallery around it; and, considering the number of years which the college has been in existence, it is remarkably poor, both in pathological specimens and anatomical preparations. What few there are of the latter are not particularly good.

There are three lecture rooms, a post-mortem room, a dissectting room (much too small), a chemical laboratory for the study of practical chemistry (also much too small), a pretty good pharmacy, a good long covered exercise ground, besides the necessary offices, forge, house surgeons' rooms, groom's rooms, etc.

* Read before the Massachusetts Veterinary Society.

There are also arrangements for hot, cold, douche and vapor baths, which were never used while I was there, and the location of which many of the students did not know; in fact, some of them did not even know of their existence.

The accommodations for patients are good, most of the stalls being boxes, well ventilated and well drained. In short, the arrangements for animals are much superior to those for students. Not that it will do for us to criticise the arrangements for students too closely, as they are much better than in any of the American schools which I have yet seen. Still, in comparison with the schools at Alfort and Berlin (the only two Continental schools which I had an opportunity of visiting) the lecture rooms, the laboratories and museum of the London school sink into insignificance.

The course of study at the English veterinary colleges comprises three years. The first year is supposed to be devoted to acquiring a knowledge of botany, chemistry and materia medica. For this purpose the student is required to attend the immense number of seven lectures a week. The result is that with so little to do, many of the young men form habits of idleness and shiftlessness, to say nothing of, in many cases, dissipation, which they never recover from.

The consequence is that there are to-day men in the London school who have been there four, five, and even six years; bright, intelligent fellows, who, if they had only been started on the right track in the beginning, and made to take up habits of studiousness and industry, would have graduated long ago with credit to themselves and their teachers.

The course for the second year comprises anatomy, histology, and physiology. To learn all this they have only two winter sessions, while for the first year's course they have to spend one summer and two winter sessions. During the second year they also have to attend clinical lectures besides receiving a certain amount of instruction in hospital practice. The last year is the hardest and best, the student having two or three lectures daily—equine practice from Prof. Robertson, cattle practice from Prof. Brown, morbid anatomy and general pathology from Prof. Axe; also lectures upon parasitology from Dr. Cobbold, all of which are very good.

In addition to the above, there are numerous clinical lectures and hospital practice, hospital practice being a sort of practical quiz given to the class in sections, in a box stall, with the horse or ox before them, by Prof. Robertson or Prof. Axe.

Then there is the cheap practice for poor people every other afternoon, where cabmen, costermongers and the like can bring their horses, ponies, donkeys, dogs, and other animals for advice and treatment, paying a nominal fee of a shilling if medicine is dispensed to them or operations performed. The latter fee should be done away with.

As the members of the senior class prescribe for or operate on these animals under the supervision of one of their teachers, and as there are often as many as thirty or forty patients present, it is an excellent opportunity for those who take the interest, to avail themselves of it, to acquire a large amount of useful, practical knowledge.

Then there are about twelve hundred horses a year examined for soundness, which the student has an opportunity of seeing and handling, too, if he desires.

Besides this, there are always from sixty to seventy horses, as well as several dogs, in the hospital, which he can observe at his leisure. Thus it will be seen that the chances for learning many things at the London school are excellent, while in other respects they are wofully deficient.

Particularly to be regretted is the time wasted the first year. This has been partially made up for recently by requiring first year students to dissect a certain amount. Still, there is lack of thorough, earnest work in the dissecting room, and the knowledge of anatomy acquired is much more superficial than it should be.

It would be a great improvement if the curriculum was changed, so as to have anatomy, histology, physiology and general chemistry taken up the first year, letting materia medica come later in the course, and having botany one of the subjects in the matriculation examination. Then there should also be a course in medical chemistry, lectures on obstetrics, and a fuller course in therapeutics; also lectures on zöology and sanitary science and police, microscopical pathology should also be gone into more deeply than at present.

This would be a great advance in veterinary education, not only in the London college, but in the other English schools as well.

Our cousins the other side of the water are so conservative and move so slowly, that it will probably be a very long time before these improvements are made.

The pupilage system struck me as a very curious one. That is, boys who wish to become veterinarians are apprenticed by their parents to veterinary surgeons for two or three years before they go to college. The consequence is that they come to college thinking that they know it all, because they can diagnose a case of colic, and know what to give for it. What causes colic and the reasons for giving certain medicines never enter their heads. The result is they do not go to work with a will, and the proportion of students plucked who have been pupils is much greater than among those who knew very little about animals before entering college. If these boys who are apprenticed to veterinary surgeons (often totally unfitted "to teach the young idea to shoot") were sent to some good school or college for the same length of time it would be much better, not only for themselves, but for the profession they are destined to adorn. It would make them more thoughtful, studious and literary, and we would not have so many complaints of the poverty of modern veterinary literature in our own language.

I do not mean to say that these young men are without education. A fair matriculation examination is required before a student can enter the college ; but at the same time there is no denying the fact that the time spent in pupilage would be much better employed at school. Some members of the profession in England, particularly the most old-fashioned ones, wish to have the pupilage system made compulsory. No student to be allowed to enter college until he has been a pupil for three years with a veterinary surgeon.

It is to be hoped that this "compulsory pupilage" clause will never be passed, and it will be a good thing when the pupilage system dies out altogether. There has been a great deal of complaint of late among the students of the hardness of the final examination. In my opinion they are fair, but not particularly hard ; no questions are asked which a veterinary surgeon should not know.

It is a great thing to have one board of examiners for all the colleges in the kingdom, so as to have the same standard at all of them. It would be a great improvement if we could have some such arrangement in this country, so that all our veterinary schools could have courses of equal length, and the same requirements for admission and graduation. Still, I am afraid that this is something never to be obtained on this continent, and diploma mills will flourish for some time to come, if not always, both in the medical profession and our own.

The fellowship degree of the Royal College of Veterinary Surgeons is a higher degree than that of members, and should be conferred as a mark of distinction for literary work, original research, or something of that kind. But under the present arrangement it is given to a man after he has been a member for five years, if he passes a written examination on various subjects and pays a fee of fifteen guineas. At a meeting of some country veterinary society (I forget just when or where, but the story is true for all that) a member got up and asked what books it was necessary to study preparatory to going up for the fellowship degree. Another member arose and told him that his check-book was the only literary production that he would need to consult.

Under the present state of affairs I should deem it no honor to be a fellow of the Royal College of Veterinary Surgeons, when acquired by undergoing an examination which any student ought to be able to pass when he graduates.

When I first began to study up the course pursued in practice by the veterinary surgeons of England, I wondered how they made a living; a country practitioner getting half-a-crown (about sixty cents) a visit, and sixpence (about twelve and a half cents) a mile for every mile he goes out of town. But I soon found out how he manages. He leaves four to six shillings worth of medicines (costing him about twopence) at every place he goes, and thus makes it up in that way. It does not matter whether the patient requires it or not; it is left. "The laborer is always worthy of his hire," and it will be a happy day for our professional brethren over there when they are better paid for their opinion and receive less for their physic. There are a few occasions when they are properly remunerated, however. There are examinations for soundness and firing, the fee for both of which is generally a guinea, or for firing two legs it is often two guineas. Professional etiquette seems to be unknown among them, or at least, I never heard of it, not even in the lecture room, where you might expect the professors to try and instil a little into the students.

I read last year in one of the veterinary periodicals of Great Britain of a case where two veterinary surgeons swore in court that a brother practitioner's charges were exorbitant, the unfortunate defendant having mulcted a farmer in the immense sum of seven shillings (about a dollar and seventy-five cents) for blistering one horse and giving his opinion on another which had something the matter with it.

But what can you expect when the head veterinary surgeon of the English army leads a magistrate aside during a recess of the court to prejudice his mind against another veterinary surgeon who was on trial. Even if this man had been so ignorant and brutal as to draw a horse's sole for side bones, it was the duty of the witness to injure him as little as possible in giving his testimony, and then to show him the folly of his ways afterwards.

The prevailing idea among the public in England regarding veterinarians is that they are simply horse and cattle doctors. Their usefulness as sanitarians has not been recognized as it should be.

Klein, Burden, Sanderson, and such men have been employed to investigate hog cholera, rinderpest, contagious pleuro-pneumonia, and similar diseases, when it should have been done by the veterinary profession. This may be partly due to the fact that there are very few men among English veterinarians capable of conducting scientific investigations. Still, I think that there are some who could do so, and I think that they have been overlooked and slighted, and have had nothing to encourage them.

One way in which a due appreciation of the English veterinarian is manifested is seen in the recognition of his services by the large agricultural societies.

At all the great agricultural societies shows—at the Smithfield fat cattle show and the cart horse show—the veterinary officer is an important individual. All animals, even pigs, are examined regarding their ages ; an animal whose mouth indicates it to be older than the class for which it is entered is disqualified. True, the veterinarian does not say it is such and such an age, but merely that its mouth indicates such and such an age. That is enough, the animal cannot compete for a prize. Horses which do not pass their examination for soundness are disqualified. Cattle, sheep and swine cannot enter the grounds until inspected and found to be free from foot and mouth disease or any other contagious malady. Neither can they be taken away after the show unless their custodian has a certificate that they are healthy, signed by the veterinary officer.

It is to be hoped that the day is not far distant when our larger agricultural societies will become civilized enough to pursue some such course. It is disgraceful to see unsound horses getting prizes over sound ones at our fairs ; three-year-old colts entered in the two-year-old class and taking the highest honors on account of their superior size ; and similar cases too numerous to mention. All our large agricultural societies, such as the New England and the various State societies, should have their veterinary officer or officers, to be on duty during their exhibitions, to examine all animals as to their age, to disqualify all unsound horses from competing for prizes, and to attend to any sick animals, free of charge to their owners.

His decision should be final ; from it there should be no appeal, and last, but not least, he should be well remunerated for his services from the funds of the society. Until within a few years there has been no legislation to regulate the practice of veterinary medicine in England, but now no one can practice as a veterinarian unless he is a member, or fellow, of the Royal College of Veterinary Surgeons, or a "registered practitioner."

A "registered practitioner" is one who has permission from the Royal College to practice as a veterinary surgeon. They are men who have been practising for a number of years, and as they will not register any young men, in time, as these old ones die off, the profession will consist entirely of members of the Royal College of Veterinary Surgeons.

This concession was necessary, in order to get protective legislation for those who were educated at college.

Now, a word about the hospital connected with the London school—how it is run on the subscription plan, and how it works. The subscription fee of the London Veterinary Hospital is two guineas a year, or twenty guineas for a life subscription. This entitles subscribers to have lame or sick animals in the hospital at a moderate weekly charge, and also to have horses examined for soundness; the fees go into the treasury of the college; the professors receive a fixed salary and are not allowed, except in very rare cases, to make a visit outside the college, and are not allowed to practice on their own account at all.

Unlike some hospitals run on the subscription plan, the London College does very little or no harm to members of the profession practicising near it. Most of its subscribers are very rich and employ their own veterinary surgeons in all ordinary cases; it is chiefly when they have horses suffering from chronic lameness that they send them to the college, after their own veterinarians fail to do them any good. In the London College you see ten cases of lameness to one of any other disease. Then their professors do not compete with other practitioners, as they are not allowed to practice outside. As for their free clinic, that is an excellent charity, although it is grumbled at by a good many veterinary surgeons in London; still, I think it does the poor more good than it does harm, besides being vastly instructive to the students who are allowed to treat these patients.

As for the schools in Scotland, I did not have a very good opportunity for investigating them. I visited both the schools in Edinburgh, but did not see the one at Glasgow.

Williams' school, for a small one, is very good, and considering that it was built by private enterprise, is large and well fitted. The Dick school is a pile of little old buildings. The most imposing thing about it is the statue of Prof. Dick in the yard.

The only Continental schools which I had an opportunity of visiting were those at Alfort and Berlin. At the former the clinic held every morning is very large, and any one in Paris can send animals there, if he chooses to pay for them.

Once a week the students perform various operations on living animals, which are afterwards killed. I was very anxious to witness these operations, but found that in order to do so I would have to get permission from the French Minister of Agriculture, and to get at him I would have had to get a letter of introduction from the United States Minister. As my time was limited I could not very well untangle the red tape necessary for the purpose.

Although France is a republic, and Germany an empire, yet I found it a much simpler matter to obtain access to the school at Berlin than to that of Alfort. I had only to ask for Dr. Lübber, and mention to him the name of Dr. Billings to be treated with the greatest kindness and courtesy, and be shown everything of interest in the institution.

I will not attempt to describe the school at Berlin more than to say that the grounds are large and handsome, well supplied with fine shade trees, and the buildings would make the professors of the best medical schools in this country turn green with envy.

Another respect in which England and the Continental countries are away ahead of us, is in the standing of their army veterinarians.

Over there, when a veterinary surgeon enters the army, he ranks as a commissioned officer, and associates with the other officers. Entering as a lieutenant, he may gradually rise to the rank of colonel.

Here, if a man is so unfortunate as to be connected with the army, he has only the rank of sergeant-major, and has only the non-commissioned officers for associates. This is a state of affairs we should all do our best to rectify as soon as possible.

Now, gentlemen, I have tried to show you in what respect our transatlantic brethren are better off than we are, and also where they are not so well off.

It is our duty to try and improve where we are remiss, and, on the other hand, where we excel we must not recede.

